# Toothache

**DOI:** 10.1192/j.eurpsy.2021.1727

**Published:** 2021-08-13

**Authors:** E. Rodríguez Vázquez, C. Capella Meseguer, J. Gonçalves Cerejeira, I. Santos Carrasco, G. Guerra Valera, M. Queipo De Llano De La Viuda, A. Gonzaga Ramírez, C. Vallecillo Adame, T. Jiménez Aparicio, C. De Andrés Lobo, A. Pérez Escudero

**Affiliations:** 1 Psiquiatría, Hospital Clínico Universitario de Valladolid, Valladolid, Spain; 2 Psiquiatría, HCUV, Valladolid, Spain; 3 Psiquiatría, Hospital Clínico Universitario Valldolid, Valladolid, Spain; 4 Psiquiatría, Hospital Clínico Universitario Valladolid, Valladolid, Spain

**Keywords:** mental disability, mental retardation

## Abstract

**Introduction:**

Mental retardation (RM) is defined as by a deficient intellectual capacity as well as by alterations of the adaptive capacity that are externalized in two or more functional areas (Personal autonomy, Communication, Orientation in the environment, Work and Free time).

**Objectives:**

Present a patient with a severe behavioural disturbance with an associated intellectual deficit, who remained hospitalized for 2 months and after observing an oral alteration her symptoms improved.

**Methods:**

A descriptive study of a clinical case

**Results:**

54-year-old woman, single. You have a moderate intellectual disability. In January 2019, she began mental health consultations with a diagnosis of adjustment disorder, on treatment with aripiprazole 5 mg/day, mirtazapine 15 mg/day, lorazepam 0.5 mg/day and dipotassium clorazepate 10 mg/day. Went to the emergency room with mutism, hyporesponsiveness and refuse to intake, having lost 25 kg in 6 months. Abdominal and thoracic CT and upper gastrointestinal endoscopy without significant findings. Consultation with otorhinolaryngology, dermatology, traumatology without significant findings. Odontostomatology consultation: Deep cavities are observed, so it is necessary to carry out extractions of the pieces in poor condition. After this intervention, the patient returns to accept oral intake.

**Conclusions:**

People with intellectual disabilities have a wide range of medical problems that in many cases are directly associated with the underlying disease or syndrome and, in others, with poor physical health due to problems in basic self-care skills or the ability to express verbally. Usually, the first manifestation of pain is an alteration in behaviour, which must be taken into account when making a differential diagnosis.

**Disclosure:**

No significant relationships.

